# Effect of Orlistat on anthropometrics and metabolic indices in children and adolescents: a systematic review and meta-analysis

**DOI:** 10.1186/s12902-023-01390-7

**Published:** 2023-07-07

**Authors:** Zeinab Nikniaz, Leila Nikniaz, Mahdieh Abbasalizad Farhangi, Hossein Mehralizadeh, Shabnam Salekzamani

**Affiliations:** 1 Liver and Gastrointestinal Diseases Research Center, Tabriz University of Medical Sciences, Tabriz, Iran; 2 Tabriz Health Services Management Research Center, Tabriz University of Medical Sciences, Tabriz, Iran; 3 Community Nutrition Department, Tabriz University of Medical Sciences, Tabriz, Iran; 4 Stem Cell Research Center, Tabriz University of Medical Sciences, Tabriz, Iran; 5 Department of Nutrition, Faculty of Health and Nutrition, Bushehr University of Medical Sciences, Bushehr, Iran

**Keywords:** Orlistat, Children, Adolescents, Metabolic Indices, Anthropometry

## Abstract

**Background:**

Childhood obesity is one of the main concerns of public health. Considering its long-term adverse health effect, various studies investigated the effect of drug therapy on anthropometric parameters and provided mixed results. In this systematic review and meta-analysis, we aimed to determine the effect of Orlistat on anthropometrics and biochemical parameters in children and adolescents.

**Materials and methods:**

The databases of PubMed, Scopus, and Web of Science were searched until September 2022. Experimental and semi-experimental studies were included if they evaluated the effect of Orlistat on obesity-related parameters in children and reported the before and after anthropometric values. A revised Cochrane risk-of-bias (Rob2) was used to evaluate the methodological quality. STATA software version 16.0 was used for the meta-analysis of the random-effect model.

**Results:**

Of 810 articles retrieved in the initial search, four experimental and two semi-experimental studies were selected for systematic review. The result of the meta-analysis of experimental studies indicated the significant effect of Orlistat on waist circumference (SMD: -0.27, 95% CI: -0.47, -0.07) and serum insulin level (SMD: -0.89, 95% CI: -1.52, 0.26). However, there were no significant effects of orlistat on body weight, body mass index, lipid profile, and serum glucose level.

**Conclusion:**

The present meta-analysis showed the significant effect of Orlistat on the reduction of waist circumference and insulin level in overweight and obese adolescents. However, due to the paucity of studies included in the meta-analysis, more prospective studies with longer duration and more sample sizes will be needed in this age group.

**Supplementary Information:**

The online version contains supplementary material available at 10.1186/s12902-023-01390-7.

## Background

Childhood obesity is considered one of the biggest concerns of public health worldwide. The prevalence of overweight and obesity is increasing in both high- and low-income countries with 340 million overweight and obese children aged more than five years old in 2016, approximately quadrupled since 1975 [1]. The rise in obesity not only increases its related complications including cardiovascular disease, hypertension, type 2 diabetes mellitus, and sleep apnea in adolescents [2] but also results in the increased risk of these non-communicable chronic diseases in adulthood [3]. In a recent Swedish prospective cohort study, obesity in childhood was shown to be the primary or contributing cause of death in more than a quarter of the deaths [4].

Within the genetic background, hypercaloric meals and sedentary lifestyles trigger obesity in children [5]. Calorie-restricted diets and exercise are the first-line strategies for the management of obesity. However, the short and long term results of these approaches in children and adolescents are even more disappointing than for adults [6].

Pharmacotherapy is another approach to the treatment of obesity in children and adolescents. Among the different drugs that were approved to use in adults, only two medications, Phentermine and Orlistat are approved by the Food and Drug Administration (FDA) for use in adolescent obesity. Orlistat is an anti-obesity drug with an inhibitory effect on intestinal lipase function. It has been reported that Orlistat results in a 30% reduction in dietary fat absorption [7]. In two systematic reviews in adults [8, 9], weight loss of 1.8 kg with Orlistat and 3.5 kg with combined Orlistat and behavioral changes were shown [9, 10]. Furthermore, a significant decrease in fasting blood glucose, total cholesterol, low-density lipoprotein cholesterol (LDL-C), triglycerides (TG), and an increase in high-density lipoprotein cholesterol (HDL-C) were found in adults taking Orlistat compared with placebo (Hanan Khalil, 2022). Moreover, the result of a clinical trial showed that orlistat and acarbose can be successfully combined in a modified-release formulation to provide efficacious weight loss with no unexpected safety issues in adults [11]. In children and adolescents, different experimental studies focused on the effect of Orlistat on children’s anthropometric characteristics and provided mixed results. Previously two systematic reviews reported the effect of Orlistat on weight and body mass index (BMI) in children. Viner et al. [12], in a systematic review of two studies, reported the modest effect of Orlistat on BMI. This systematic review was conducted in 2010 and since then several additional experimental studies have been published. Another systematic review only included studies with an intervention duration of more than six months and showed that among different anti-obesity drugs, only Liraglutide had a clinically significant effect on weight loss [13]. Considering that most experimental studies that assess the effect of Orlistat on anthropometric factors were assessed for its shorter duration, it seems that there is a need for an updated systematic review that considered all studies that assess the effect of Orlistat on weight loss in children. In this systematic review, we aimed to determine the effect of Orlistat on weight, BMI, lipid profile, fasting blood glucose, and insulin in children and adolescents.

## Materials and methods

The Preferred Reporting Items for Systematic Review and Meta-analysis (PRISMA) were used for reporting this systematic review and meta-analysis study. The study was registered in PROSPERO (CRD42022383212).

### Search strategy

Two researchers searched the databases of PubMed, Scopus, and Web of Science for September 2022 independently. The keywords were designated based on the Population (children), and intervention (Orlistat). Table S1 presented the thorough search strategy for each database. We also searched the reference list of the related articles for more relevant articles.

One reviewer (ShS) omitted duplicate articles and then two reviewers (ShS and ZN) independently screened the records by title and abstract according to the study aim, included population, and intervention. For the remaining articles, full papers were obtained and were assessed according to inclusion/exclusion criteria. Any discrepancies were resolved through discussion and by consulting a third reviewer (LN).

#### Inclusion criteria

Experimental and semi-experimental that evaluated the effect of Orlistat on obesity-related parameters in children and reported the before and after values of studied parameters were included. Only experimental studies were entered into the meta-analysis. Case reports, case series, letters, review articles, and studies published only in abstract form were excluded. We did not included the studies that use another drug alongside Orlistat for weight loss. There were no restrictions regarding language.

### Outcomes

The primary outcomes of the present study were the effect of Orlistat on anthropometric parameters in children. The secondary outcomes were the effect of Orlistat on lipid profile, glucose, and insulin level in children.

### Assessment of risk of bias

A revised Cochrane risk-of-bias (Rob2) and the respective excel application was used to evaluate the methodological quality of the studies. For each study, the following parameters were assessed: randomization, deviations from the intended interventions, missing outcome data, the measurement of the outcome, and the selection of the reported result. According to this tool guideline, studies were classified as a low risk of bias, some concerns relating to the risk of bias, or a high risk of bias. Two reviewers independently evaluate each paper (ShS and ZN) and discrepancies were resolved by discussion and consensus by a third person (MAF).

### Data extraction

Two authors (ShS and ZN) extracted predefined information independently. The author-designed extraction form was used in this regard. The following information was extracted: the first author’s name and publication date, country, study region, design, age group, sample size, and outcomes. Any discrepancies were resolved through discussion.

### Statistical analysis

STATA software version 16.0 was used for the meta-analysis of the random-effect model. For the meta-analysis, the mean changes of studied parameters, and sample size of each group were extracted and pooled using a random-effect model. Two experimental studies reported the studied parameters in two-time intervals (three and six months) [14, 15], and were entered into the meta-analysis separately. When three or more studies were available and heterogeneity was high (I^2^ > 75% and p-value of < 0.05) [16], the subgroup meta-analysis was conducted. In the case of medians and ranges or 95% confidence intervals [CIs], mean and standard deviation (SD) values were estimated using Hozo et al. method. We estimated the SD for mean change from the baseline to the endpoint by averaging the calculated correlation coefficients. Subgroup analysis was done considering that the effect of Orlistat on studied parameters may differ according to intervention duration, and other lifestyle modifications such as exercise, dietary recommendations, and multivitamin use. Any potential publication bias was identified using the funnel plot and with Begg’s rank correlation and Egger’s weighted regression tests. For adjusting the analysis of the effects of publication bias, we used the Duval & Tweedie “trim and fill” method [17]. A probability value (p-value) < 0.05 was considered statistically significant.

## Results

**Fig. 1:**
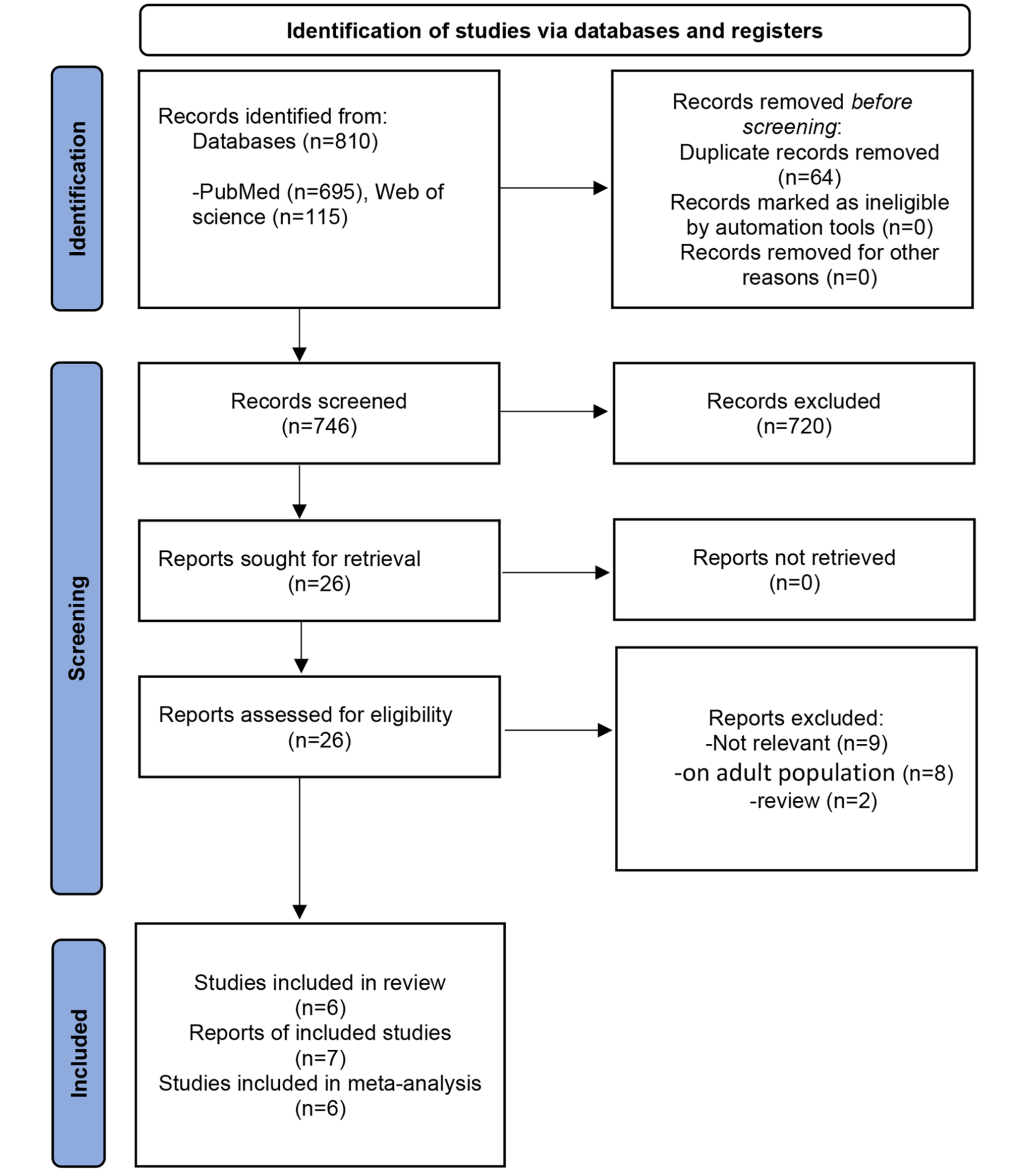
PRISMA flow diagram

Of 810 articles retrieved in the initial search, 62 articles were excluded as duplicates, and 724 were excluded in the title/abstract evaluation phase. Finally, four experimental studies and two semi-experimental studies (including three papers) were selected for systematic review. Only experimental studies were entered into the meta-analysis. The flow chart of studies inclusion is provided in Fig. 1.

### Systematic review result

Table 1 presents the characteristics of included studies. Six out of seven included studies were published between 2002 and 2006. Two studies were conducted in Asia [14, 18], one in Europe [19], and four in America [15, 20, 21]. Two studies had semi-experimental before-after designs [19, 20], and four had randomized controlled clinical trials (RCT) designs. All except one [21] had fewer than 50 participants. Orlistat dose was consistent in all studies (120 mg, three times a day), however, the intervention duration was varied from 10 weeks to 52 weeks.

**Table 1:** Characteristics of included studies

Author (date)	Country	Study Design	Means Age	Sample size		Trial Duration (Week)	Dose	Intervention	
				Intervention group	Control group			Intervention group	Control group
Quasi-experimental studies									
Norgren et al. (2003)	Sweden	BA	7 to 12	11	-	12	120 mg tid	Orlistat dietary information	-
McDuffie et al. (2002)	United States	BA	12 to 17	20	-	12	120 mg tid	-Orlistat -Fat soulable vitamins (5000 IU of vitamin A (80% as retinol acetate; 20% as -carotene), 400 IU of vitamin D (as ergocalciferol), 30 IU of vitamin E (as DL–tocopheryl acetate), and 25 g of vitamin K1 (as phytonadione) -Energy–restricted diet (500-kcaldeficit diet containing no more than 30% of calories from fat) -Exercise program (30 min of daily aerobic exercise and inclusion of lifestyle exercise whenever possible, monitored by pedometer readings) -Behavior modification (stimulus control and eating-management skills)	-
McDuffie et al. (2004)	United States	BA	12 to 17	20	-	24	120 mg tid	-Orlistat -Fat soulable vitamins (5000 IU of vitamin A (80% as retinol acetate; 20% as -carotene), 400 IU of vitamin D (as ergocalciferol), 30 IU of vitamin E (as DL–tocopheryl acetate), and 25 g of vitamin K1 (as phytonadione) -Energy–restricted diet (500-kcaldeficit diet containing no more than 30% of calories from fat) -Exercise program (30 min of daily aerobic exercise and inclusion of lifestyle exercise whenever possible, monitored by pedometer readings) -Behavior modification (stimulus control and eating-management skills)	
Randomized clinical trials									
Ozkan et al. (2004)	Turkey	RCT	10–16	15	15	12	120 mg tid	Orlistat diet (20% reduction in daily calories calculated for age and sex) exercise (at least 30 min of moderate exercise per day) daily oral multivitamin	diet (20% reduction in daily calories calculated for age and sex) exercise (at least 30 min of moderate exercise per day) daily oral multivitamin
Yu et al. (2013)	Japan	RCT	11 to 18	21	20	10	120 mg tid	Orlistat Dietary supervision (30% reduction in calorie intake based on dietary record. The menu, low in fat (25–30%), high in complex carbohydrate (55–65%) and sufficient in protein (15–20%) to support growth) Exercise (resistance training twice a week, with each session lasting 70 min. multivitamin (A Multivitamin supplement of fat soluble vitamins (5000 IU of vitamin A, 400 IU of vitamin D, 30 IU of vitamin E and 25 mg of vitamin K)	Dietary supervision (30% reduction in calorie intake based on dietary record. The menu, low in fat (25–30%), high in complex carbohydrate (55–65%) and sufficient in protein (15–20%) to support growth) Exercise (resistance training twice a week, with each session lasting 70 min. multivitamin (A Multivitamin supplement of fat soluble vitamins (5000 IU of vitamin A, 400 IU of vitamin D, 30 IU of vitamin E and 25 mg of vitamin K)
Chanoine et al. (2005)	Canada and US	RCT	12 to 16	352	181	52	120 mg tid/	-Orlistat -Hypocaloric diet (The caloric intake was calculated to provide a reduction in estimated caloric requirements of approximately 40%) -multivitamin -guidelines for diet exercise behavioral modification	Placebo and -hypocaloric diet (The caloric intake was calculated to provide a reduction in estimated caloric requirements of approximately 40%) -multivitamin -guidelines for diet exercise behavioral modification
Maahs et al. (2006)	Mexico	RCT	14 to 18	20	20	12	120 mg tid /placebo	Orlistat Hypocaloric diet (subtracted 500 calories from expected calorie need calculated by Harris-Benedict equation) pediatric activity pyramid (at least 3 times per week for at least 30 min)	Hypocaloric diet (subtracted 500 calories from expected calorie need calculated by Harris-Benedict equation) pediatric activity pyramid (at least 3 times per week for at least 30 min)

Two studies were conducted with quasi-experimental design. Mcduffie et al. [20] showed a significant reduction in weight (3.5 ± 6.0%), waist circumference, and BMI after six months of Orlistat intervention. Another before-after study also reported a significant reduction in body weight and BMI after 12 weeks of Orlistat intervention [19].

Four studies were conducted with the RCT design. One had an open-labeled design [18]. Maahs et al. [15] measured the outcomes after three, and six months. Yu et al. [14] had two interventions group namely the “Orlistat and low-calorie diet” group and the “Orlistat, low-calorie diet, and exercise” group. The outcome of these different groups was entered into the meta-analysis separately. All except one [15] showed a significant reduction in weight and BMI in the Orlistat group compared with the control group.

### Result of risk of bias

**Fig. 2:**
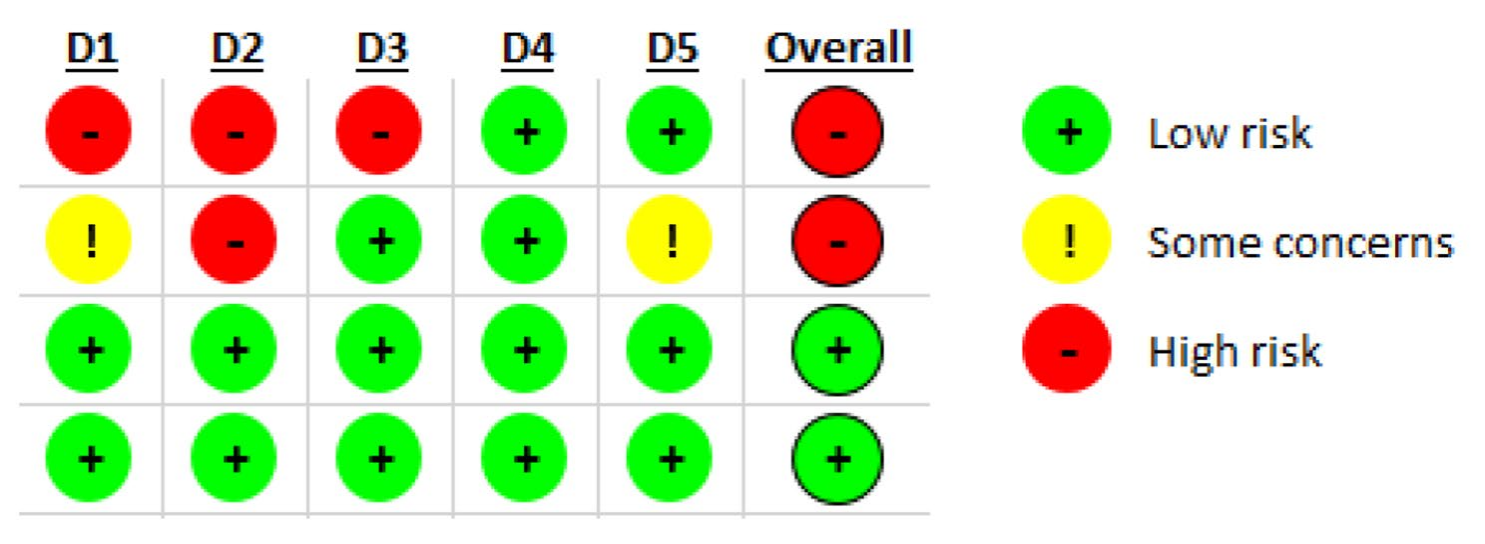
The result of risk of bias. D1: Randomization process; D2: Deviations from the intended interventions; D3: Missing outcome data; D4: Measurement of the outcome; D5: Selection of the reported result

Figure 2 presents the result of the risk of bias. As can be seen, two studies had a high risk of bias due to not being randomized [14, 18], not double blinded [14, 18], and having missing outcome data [18].

### Meta-analysis result

**Fig. 3:**
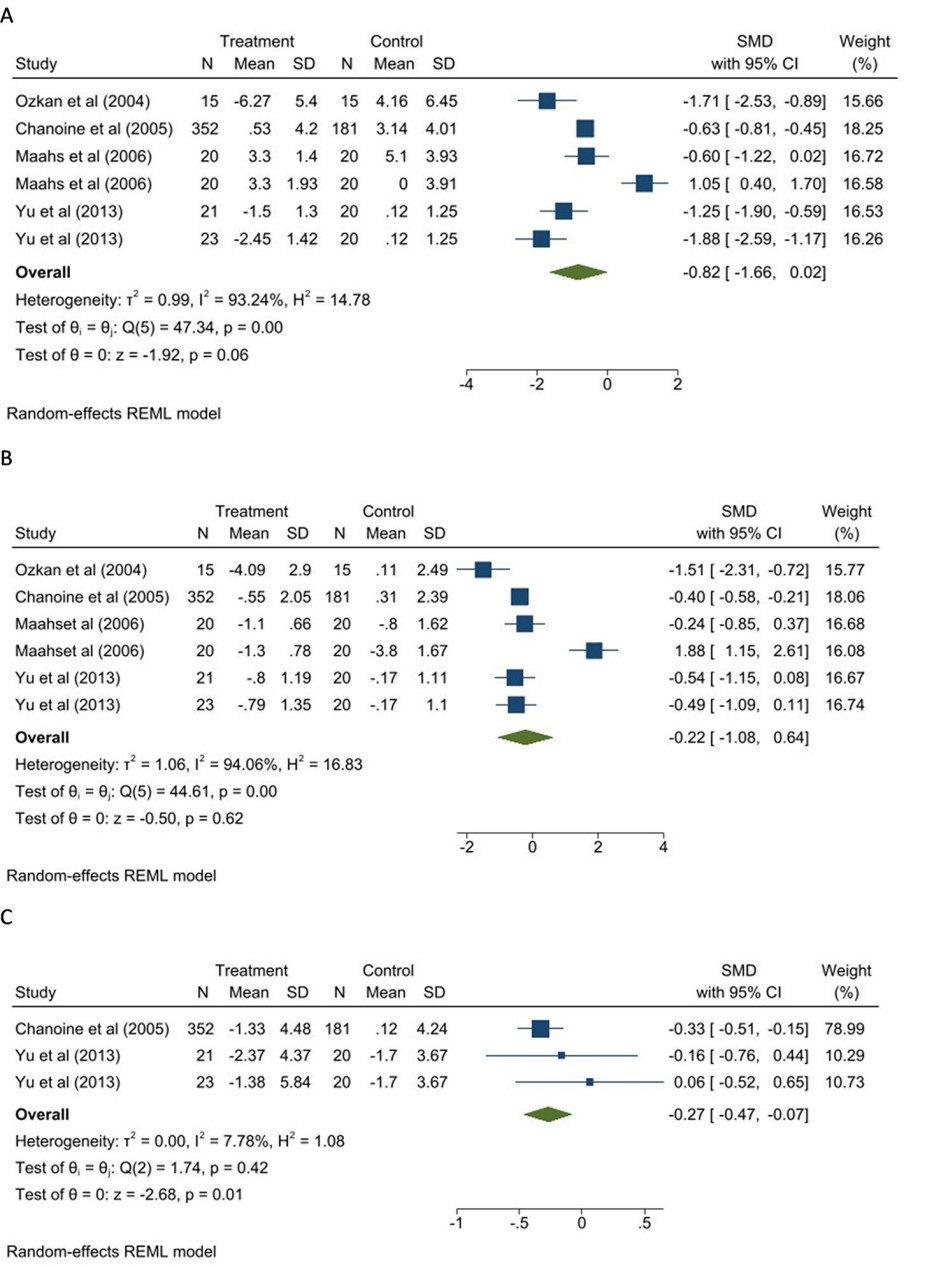
The effect Orlistat on **A**: weight, **B**: body mass index;**C**: waist circumference

The meta-analysis of the effect of the Orlistat on anthropometric measurements is shown in Fig. 3. As can be seen in Fig. 3A, four studies (including six reports) compared the weight changes between the Orlistat group and the control group. Considering that the high heterogeneity (I^2^: 93.24%) was observed between studies, the random-effect model was used, which found an insignificant effect of Orlistat on weight reduction (SMD: -0.82, 95% CI: -1.66, 0.02).

Subgroup analysis indicated that interventions with a duration of fewer than three months (p = 0.001), and Orlistat intervention along with multivitamin use (p < 0.001) had a significant effect on weight. The test of group differences indicated no significant differences between groups (Figure S1A). Considering the low number of studies in exercise and diet subgroups (less than 2), no subgroup analysis were performed for these variables.

The result of funnel plot (Figure S2A) and egger test (P = 0.58) showed no evidence of publication bias.

The meta-analysis of the result of four studies (six reports) that evaluated the effect of Orlistat on BMI is shown in Fig. 3B. Due to the high heterogeneity observed between studies (I^2^: 94.06%), the random-effect model was used. The result showed insignificant differences in BMI between the Orlistat group and the control group (SMD: -0.22, 95% CI: -1.08, 0.64).

The subgroup analysis indicated that interventions with a duration of fewer than three months (p = 0.01), and Orlistat intervention along with multivitamin use (p = 0.002) had a significant effect on BMI with no significant differences between groups (Figure S1B). Considering the low number of studies in exercise and diet subgroups (less than 2), no subgroup analysis were performed for these variables.

The result of funnel plot (Figure S2B) and egger test (p = 0.85) showed no evidence of publication bias.

The two studies (three reports) that reported waist circumference were entered into the meta-analysis (Fig. 3C) and the result showed a significant effect of Orlistat on this parameters (SMD: -0.27, 95% CI: -0.47, -0.07).

Considering that the number of studies in some subgroups were less than 2, no subgroup analysis were performed.

The result of funnel plot (Figure S2C) and egger test (p = 0.23) showed no evidence of publication bias.

**Fig. 4:**
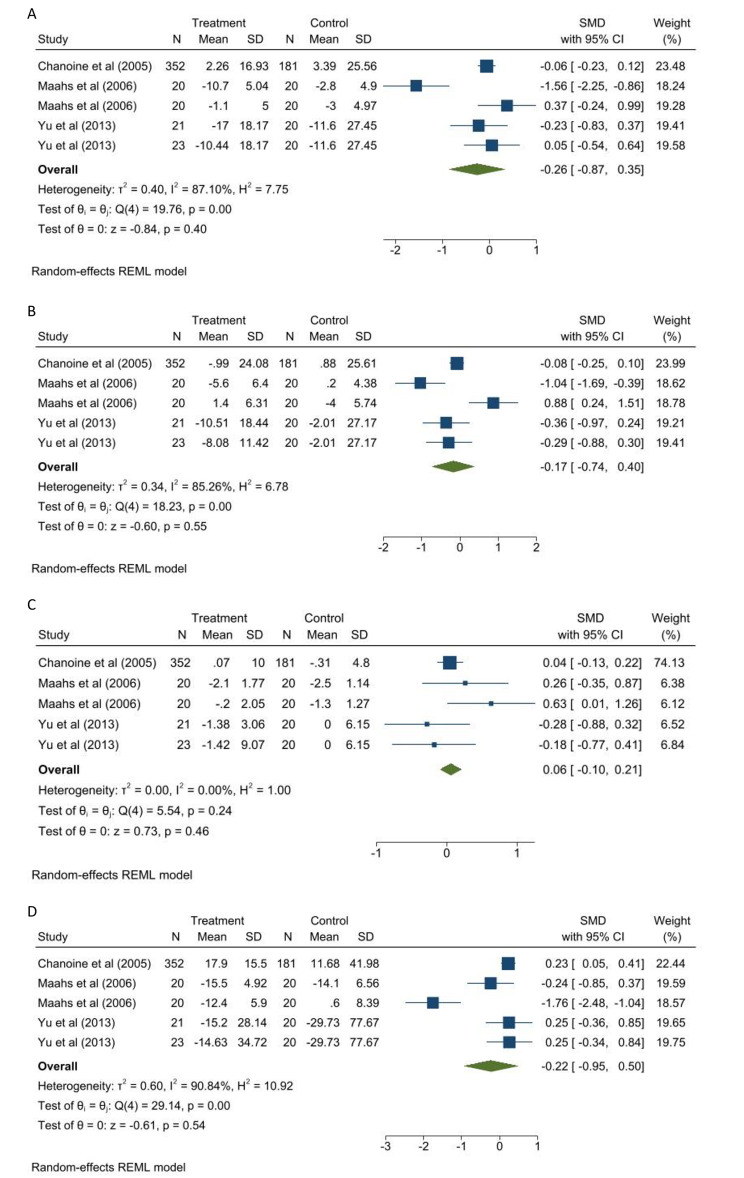
The effect Orlistat on **A**: Total cholesterol, **B**: LDL-C; **C**: HDL-C; **D**: triglyceride

Figure 4 depicts the result of the meta-analysis regarding the effect of Orlistat on lipid profile. Three studies (five reports) were entered into the meta-analysis and the random-effect model indicated no significant effect of Orlistat on serum total cholesterol (Fig. 4A), LDL-C (Fig. 4B), HDL-C (Fig. 4C), and TG (Fig. 4D).

Subgroup analysis confirmed that interventions with a duration of fewer than three months (p = 0.01) had a significant effect on serum cholesterol levels. Orlistat intervention without multivitamin use (p = 0.04) had a significant effect on serum HDL-C and Orlistat intervention along with multivitamin use had a significant effect on serum TG level (p = 0.006). However, there were no significant differences between groups (Figure S3A-D). Considering the low number of studies in exercise and diet subgroups (less than 2), no subgroup analysis were performed for these variables.

The result of funnel plot (Figures S4A-D) and egger test (p > 0.05) showed no evidence of publication bias.

**Fig. 5:**
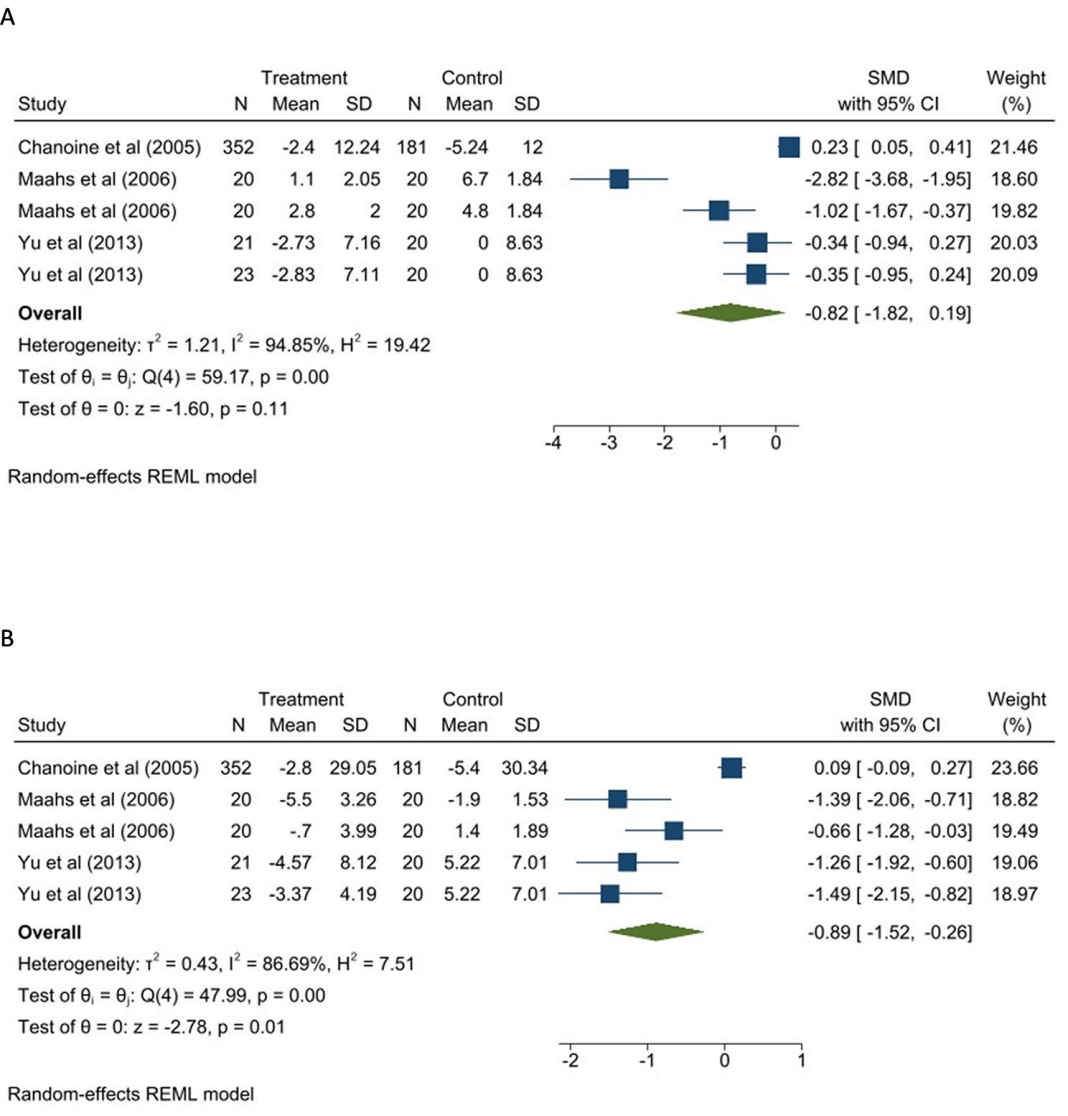
The effect Orlistat on **A**: serum Glucose, **B**: insulin

Three studies (five reports) were entered in the meta-analysis of the effect of Orlistat on serum glucose and insulin levels (Fig. 5). Due to the high heterogeneity observed between studies (I^2^: 86.69%), the random-effect model was used. As depicted in Fig. 5A, differences in serum glucose between the Orlistat group and control group (SMD: -0.82, 95% CI: -1.82, 0.19) were insignificant.

The random-effect model meta-analysis indicated significant effects of Orlistat on serum insulin level (SMD: -0.89, 95% CI: -1.52, 0.26) (Fig. 5B).

Subgroup analysis confirmed that Orlistat with multivitamin use had a significant effect on serum glucose levels (p = 0.03) (Figure S5A). Interventions with a duration of fewer than three months (p < 0.001), and with a multivitamin (p = 0.005) significantly lowered serum insulin levels, with no significant differences between groups (Figure S5B). Considering the low number of studies in exercise and diet subgroups (less than 2), no subgroup analysis were performed for these variables.

The result of funnel plot (Figure S6) and egger test (p < 0.05) showed evidence of asymmetric distribution of studies and small study effect for glucose and insulin. But after trim and fill analysis, the glucose (no imputed study) and insulin (one imputed study) remained unchanged.

## Discussion

Pharmacological treatments along with diet and behavioral therapy are the available interventions for the treatment of obesity in children and adolescents. In this regard, different studies have assessed the effect of Orlistat on weight loss in children and adolescents and reported diverse results. The meta-analysis of the result of these studies showed that Orlistat had a beneficial effect on waist circumference and insulin levels in children and adolescents. However, effects of Orlistat on weight, BMI, blood glucose level, and lipid profile, while beneficial, were insignificant.

Contrary to our result, in the meta-analysis of studies conducted with Orlistat in adults, showed mean weight loss of 2.89 Kg in 12 months [22]. Orlistat has been also shown to be an effective anti-obesity agent in overweight and obese adults for up to four years [23–25]. In the present meta-analysis, Chanione et al. [21] had the longest duration of intervention (52 weeks) showed an early decrease of weight in 12 weeks; however, at the end of the study, the weight change was only 0.53 Kg.

Only two studies reported a significant reduction in weight by the use of Orlistat [14, 18]. The largest mean weight reduction 6.27 kg in the Orlistat-treatment group after 12 weeks was reported by Ozkan et al. [18]. Of note treatment of obesity during growth and development should be monitored carefully so to not impair normal growth.

There was a marginal decrease in waist circumference in the Orlistat group compared to the placebo group (0.27 cm). Only two studies were included in the analysis of waist circumference [14, 21], making the interpretation difficult. In a recent meta-analysis on overweight and obese adults, Orlistat caused a significant reduction of 6.96 cm in waist circumference in six months [26]. Another meta-analysis study, it was shown a significant reduction in waist circumference in women with Polycystic ovary syndrome [27]. No study adjusted the change in waist circumference for the change in BMI, such as with a Body Shape Index, which normalizes waist circumference for height and weight [28].

In this meta-analysis, Orlistat provided insignificant reductions in lipid profile and glucose concentration. A previous meta-analysis of thirty-three RCTs showed that Orlistat did significantly reduced the concentration of cholesterol, triglyceride, and LDL-C and increased HDL-C levels in obese and overweight adults [29]. In those study, the mean lipid concentrations were elevated; in contrast, adolescents usually do not have elevated lipids, which may explain the non-significant effect of Orlistat in this group.

In the present meta-analysis of overweight and obese adolescents, Orlistat caused a significant reduction in insulin levels of 0.89 µIU/mL. In a study of patients with type 2 diabetes, Orlistat improved insulin concentration after six months [30]. The effect of Orlistat on insulin might be attributed to the weight loss consequences of Orlistat [31] which resulted in an improvement in glucose hemostasis [32].

The results of the present study should be interpreted cautiously, considering the several limitations. Foremost is the limited number, small size, and short duration of RCTs available for inclusion in the meta-analysis. However, considering that there were no limitations regarding language, date, dose, duration of intervention, and region, our search method was comprehensive. Moreover, the results indicated some heterogeneity. So, a subgroup analysis was conducted. Considering the limitations of the included studies, prospective studies with longer duration and larger sample sizes will be need to be conducted to determine the role of Orlistat in treating overweight and obese adolescents. In addition considering the presence of drug-drug interaction of orlistat with hydrolases, future trials should consider this issue [33].

## Conclusion

In conclusion, the present meta-analysis showed the significant effect of Orlistat on the reduction of waist circumference and insulin level in overweight and obese adolescents. However, reductions in weight, BMI, and lipid profile with Orlistat were insignificant.

## Electronic supplementary material

Below is the link to the electronic supplementary material.

Supplementary Material 1

Supplementary Material 2

## Data Availability

The datasets generated and/or analyzed during the current study are not publicly available due to the institution’s policy, but are available from the corresponding author upon reasonable request.
