# Supplementary material for: Effect of Orlistat on anthropometrics and metabolic indices in children and adolescents: a systematic review and meta-analysis
Source: BMC Endocr Disord. 2023 Jul 7;23:142. doi: 10.1186/s12902-023-01390-7 (PMC10327388; doi:10.1186/s12902-023-01390-7)
Supplement: Supplementary file 1 — Supplementary Material 1 [file 12902_2023_1390_MOESM1_ESM.docx]

Table S1: Search strategy

| database | Search strategy | result |
| --- | --- | --- |
| PubMed  Date: Sep 10 2022 | Search: ((((((((((((((((((((((Pediatrics[Title/Abstract])) OR (Pediatrics[Other Term])) OR (child[MeSH Terms])) OR (child*[Title/Abstract])) OR (child*[Other Term])) OR (Child Preschool[MeSH Terms])) OR (Adolescent[MeSH Terms])) OR (Adolescent*[Title/Abstract])) OR (Adolescent*[Other Term])) OR (Teen*[Other Term])) OR (Teen*[Title/Abstract])) OR (Youth*[Title/Abstract])) OR (Youth*[Other Term])") OR (pediatrics[MeSH Terms])) OR (pediatrics[MeSH Terms])) OR (Adolescence[Other Term])) OR (Adolescence[Title/Abstract])) AND (((((((((((((((((((((((((((((Pediatric Obesity[MeSH Terms]) OR (obesity[MeSH Terms])) OR (overweight[MeSH Terms])) OR (overnutrition[MeSH Terms])) OR (body weight[MeSH Terms])) OR (pediatric obesity[Title/Abstract])) OR (pediatric obesity[Other Term])) OR (obesity[Other Term])) OR (obesity[Title/Abstract])) OR (overweight[Title/Abstract])) OR (overweight[Other Term])) OR (overnutrition[Other Term])) OR (overnutrition[Title/Abstract])) OR (body weight[Title/Abstract])) OR (body weight[Other Term])) OR (Obesity in Childhood[Other Term])) OR (Obesity in Childhood[Title/Abstract])) OR (Childhood Onset Obesity[Title/Abstract])) OR (Childhood Onset Obesity[Other Term])) OR (Child Obesity[Other Term])) OR (Child Obesity[Title/Abstract])) OR (Childhood Obesity[Title/Abstract])) OR (Childhood Obesity[Other Term])) OR (Hypernutrition[Other Term])) OR (Hypernutrition[Title/Abstract])) OR (adolescents obesity[Other Term])) OR (adolescents obesity[Title/Abstract])) OR (adolescents overweight[Title/Abstract])) OR (adolescents overweight[Other Term]))) AND ((((((((((((((((((((((Orlistat[MeSH Terms]) OR (Orlistat[Title/Abstract])) OR (Orlistat[Other Term])) OR (Tetrahydrolipstatin[Other Term])) OR (Tetrahydrolipstatin[Title/Abstract])) OR (THLP[Title/Abstract])) OR (THLP[Other Term])) OR (Tetrahydrolipastatin[Other Term])) OR (Tetrahydrolipastatin[Title/Abstract])) OR (Ro-18-0647[Title/Abstract])) OR (Ro-18-0647[Other Term])) OR (Ro 18 0647[Other Term])) OR (Ro 18 0647[Title/Abstract])) OR (Alli[Title/Abstract])) OR (Alli[Other Term])) OR (1-((3-Hexyl-4-oxo-2-oxetanyl)methyl)dodecyl-2-formamido-4-methylvalerate[Other Term])) OR (1-((3-Hexyl-4-oxo-2-oxetanyl)methyl)dodecyl-2-formamido-4-methylvalerate[Title/Abstract])) OR (Xenical[Title/Abstract])) OR (Xenical[Other Term])) OR (Anti-Obesity Agents[MeSH Terms])) OR (Anti-Obesity Agents[Title/Abstract])) OR (Anti-Obesity Agents[Other Term])) | 695 |
| Web of Science  Date: Sep 10 2022 | 1: TS=("Pediatric Obesity "OR overweight OR body weight OR body weight OR "Obesity in Childhood" OR "Childhood Onset Obesity" OR "Child Obesity[Other Term" OR "Childhood Obesity" OR Hypernutrition OR "adolescents obesity" OR "adolescents overweight")  2: TS=(Orlistat OR Tetrahydrolipstatin OR THLP OR Tetrahydrolipastatin OR Ro-18-0647 OR "Ro 18 0647" OR Alli OR 1-((3-Hexyl-4-oxo-2-oxetanyl)methyl)dodecyl-2-formamido-4-methylvalerate OR Xenical OR "Anti-Obesity Agents" OR "anti obesity agent")  3: TS=(Pediatrics OR child* OR Adolescent OR Teen* OR Youth* OR pediatrics OR Adolescence)  4: #1 AND #2 AND #3 | 115 |
