# Supplementary material for: Effect of Orlistat on anthropometrics and metabolic indices in children and adolescents: a systematic review and meta-analysis
Source: BMC Endocr Disord. 2023 Jul 7;23:142. doi: 10.1186/s12902-023-01390-7 (PMC10327388; doi:10.1186/s12902-023-01390-7)
Supplement: Supplementary file 2 — Supplementary Material 2 [file 12902_2023_1390_MOESM2_ESM.docx]

A:


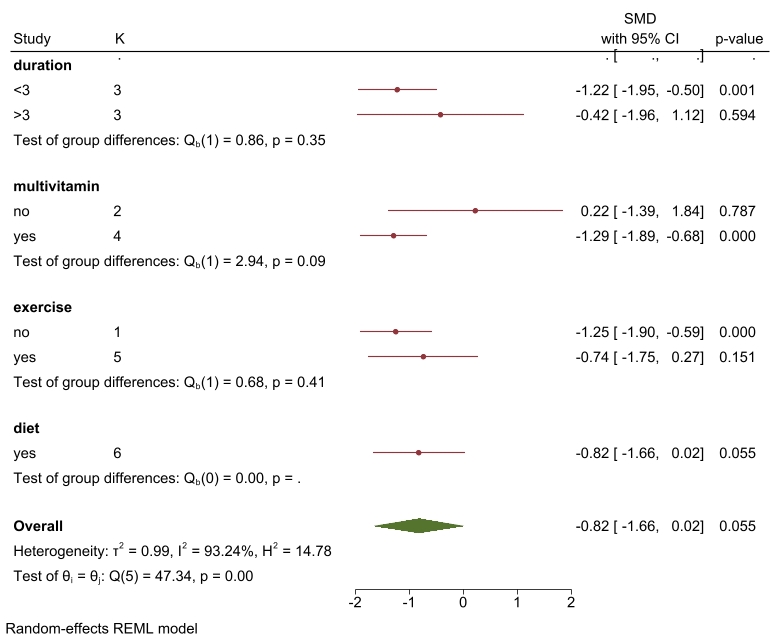


B:


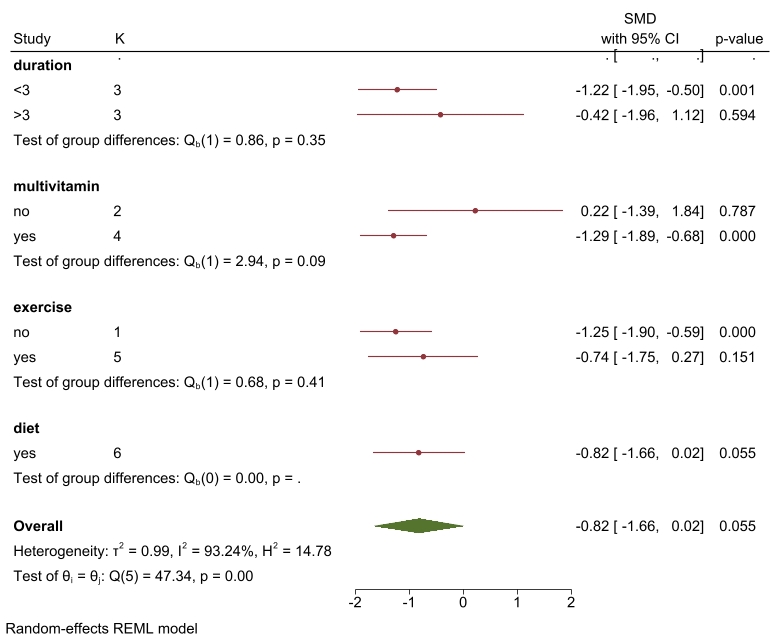


C


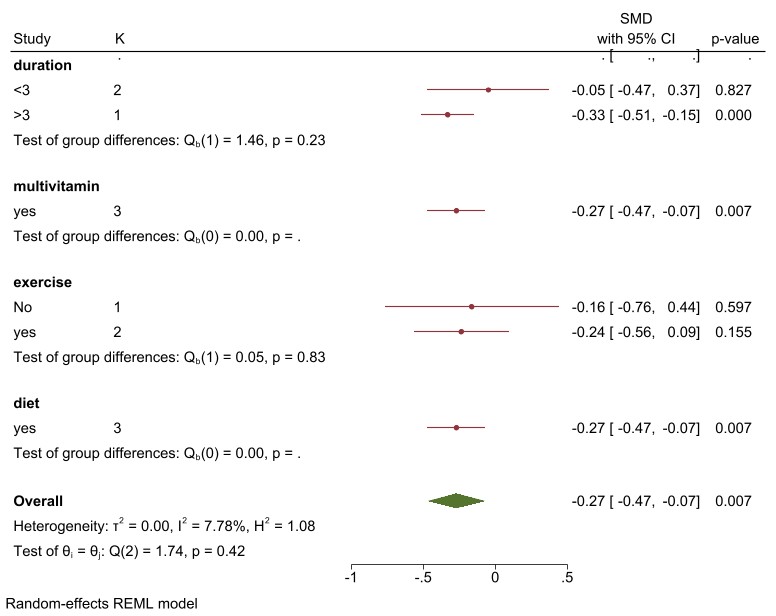


Figure S1: the effect Orlistat on A: weight, B: body mass index; C: waist circumference based on different subgroup analysis.

A


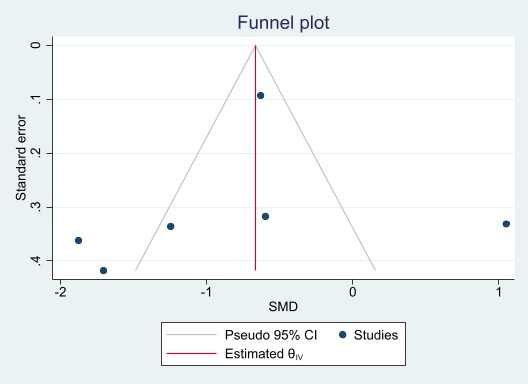


B


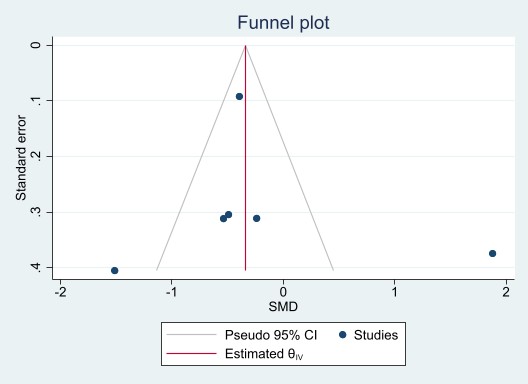


C


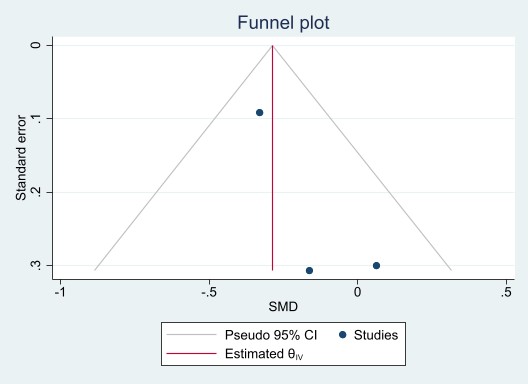


Figure S2: the funnel plot of the effect Orlistat on A: weight, B: body mass index; C: waist circumference

A


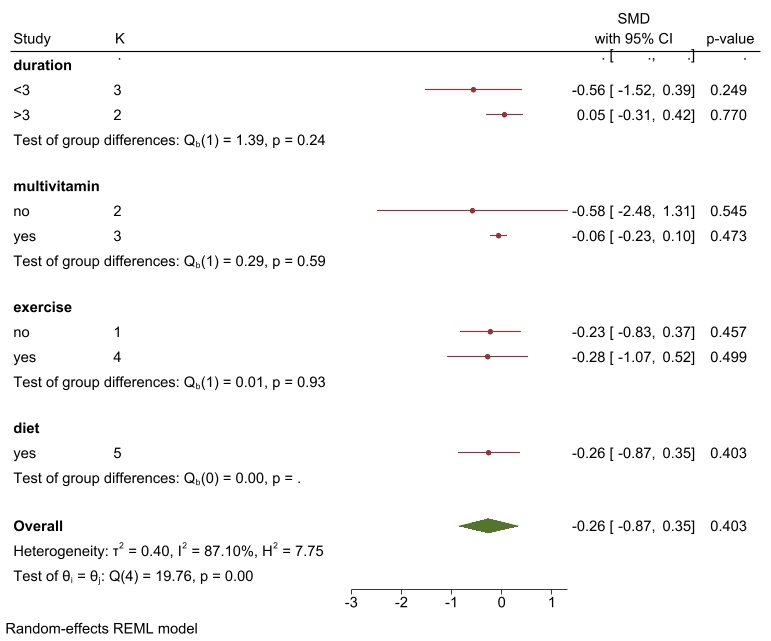


B


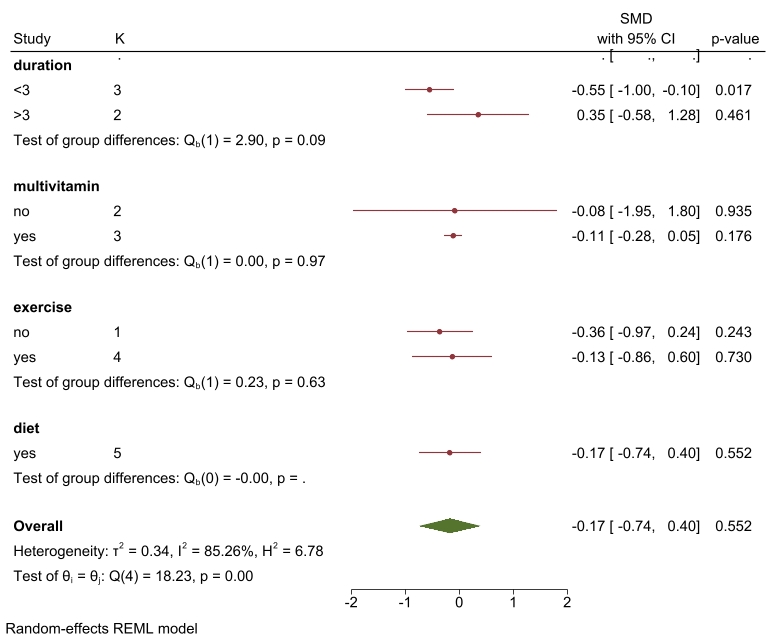


C:


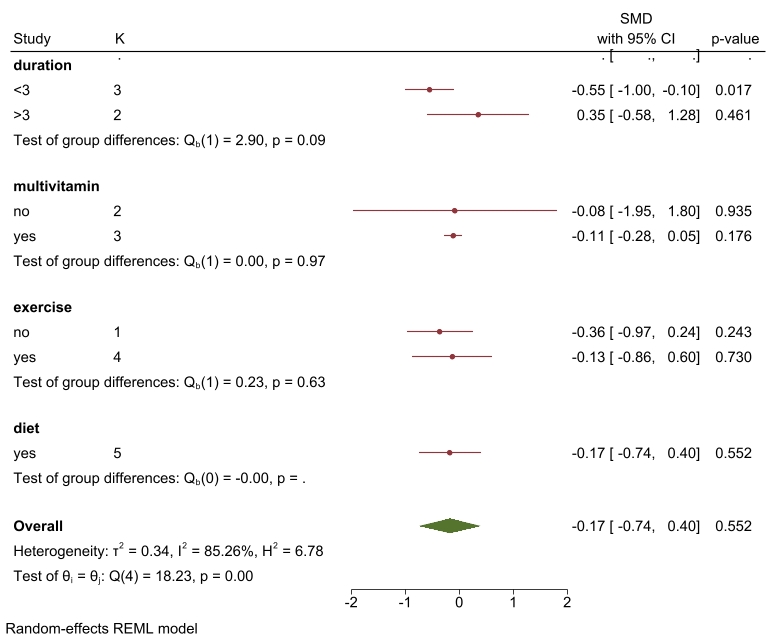


D:


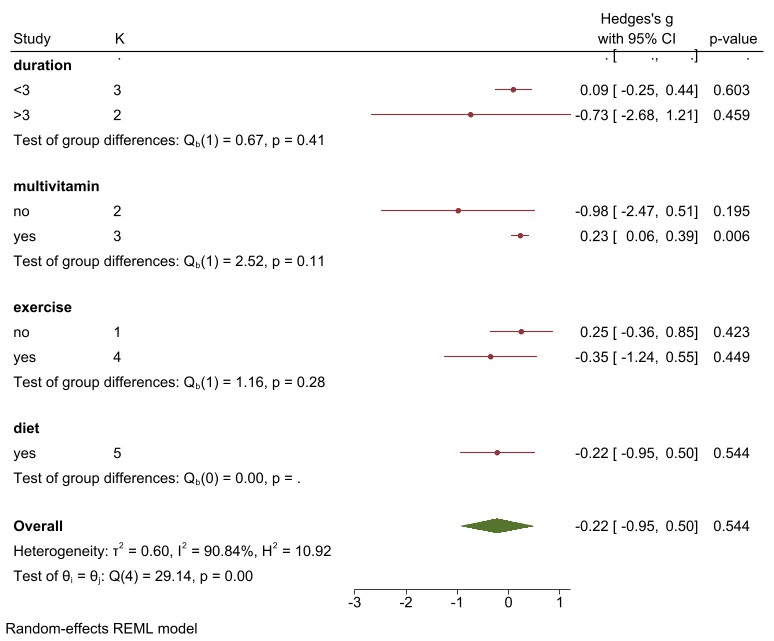


Figure S3: the effect Orlistat on A: total cholesterol, B: LDL-C; C: HDL-C, D: triglyceride based on different subgroup analysis.

A


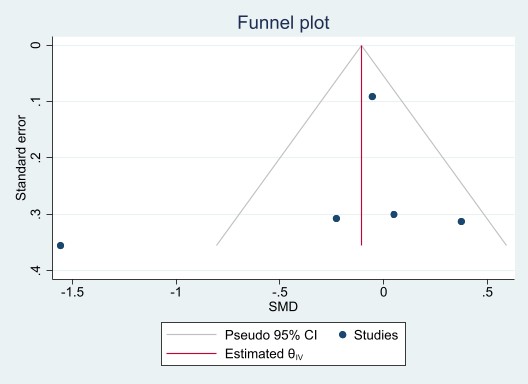


B


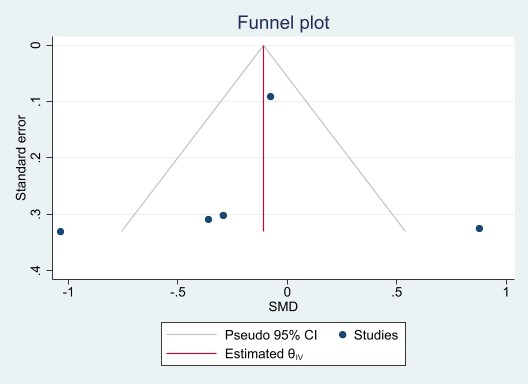


C


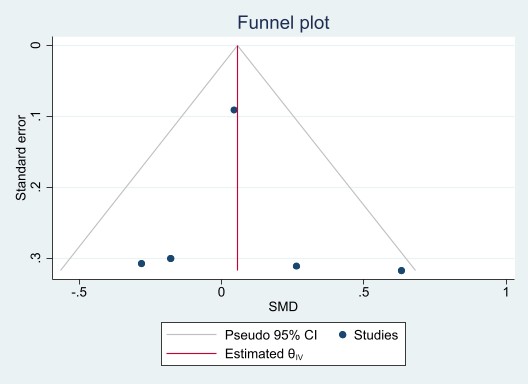


D


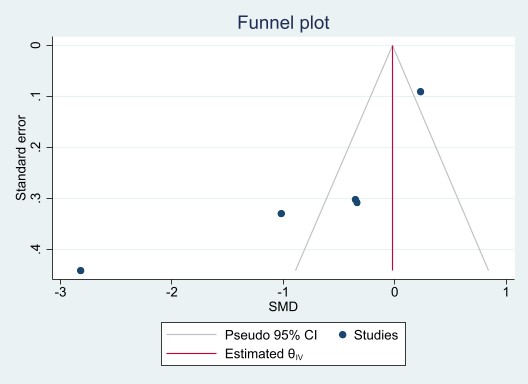


Figure S4: The funnel plot of the effect Orlistat on A: Total cholesterol, B: LDL-C; C: HDL-C; D: triglyceride

A:


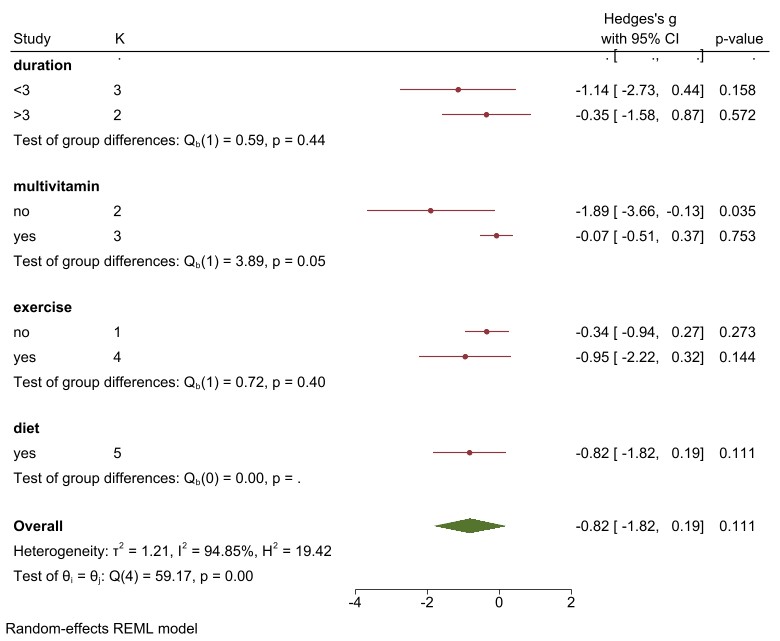


B:


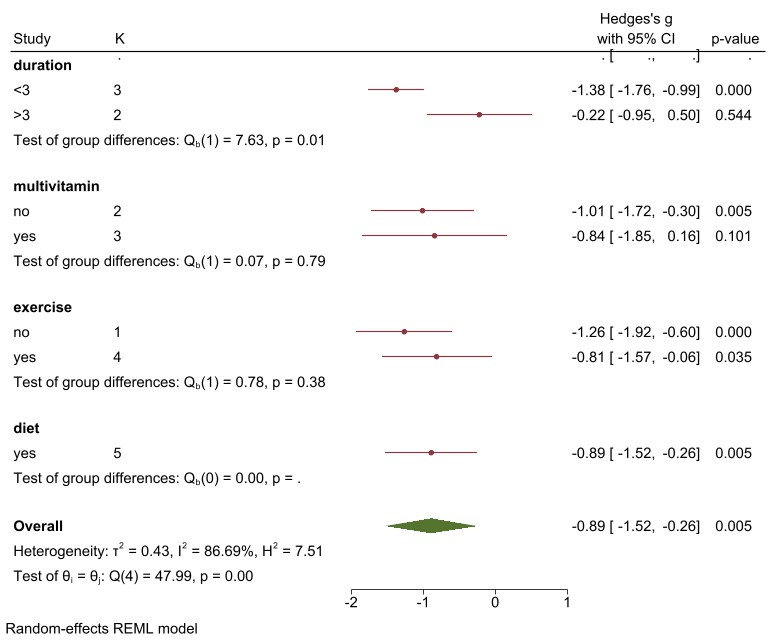


Figure S5: the effect Orlistat on A: glucose, B: insulin based on different subgroup analysis.

A:


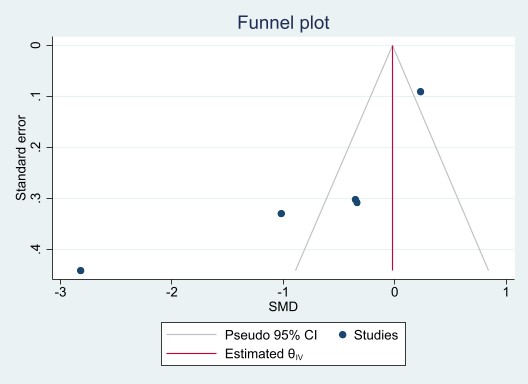


B:


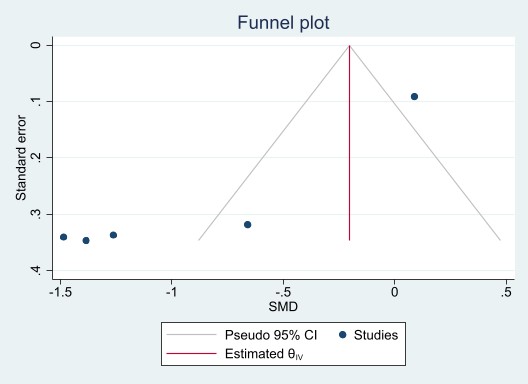


Figure S6: The funnel plot of the effect Orlistat on A: serum Glucose, B: insulin
